# Supplementary material for: Quantitative Conspicuity of Pancreatic Canine Insulinoma: A Comparison of Dynamic 4D CT and Dual-Source, Dual-Energy Bolus-Triggered Multiphase CT Imaging
Source: Vet Sci. 2025 Nov 19;12(11):1102. doi: 10.3390/vetsci12111102 (PMC12656957; doi:10.3390/vetsci12111102)
Supplement: Supplementary file 1 [file vetsci-12-01102-s001.zip › vetsci-3849417-supplementary.pdf]

Table S1: Glucose values, serum insulin, type of diagnosis, tumor localization.

PATIENT n.	Glusose (mg/dL) - Reference Range 81-112 mg/dL	Insuline (µU/mL)	Diagnosis (H: hisological, CY: Cytological, CL:clinical)	Tumor localization on CT - if multiple lesions are present, the largest is indicated (R: right pancreatic lobe; B: body of the pancreas; L: left pancreatic lobe)
1	75	57.4	H	L
2	31	60.1	CY	R
3	43	27.0	H	L
4	67	10.6	H	L
5	39	29.8	Cl	L
6	18	12.5	Cl	B
7	46	43.2	H	L
8	43	27.0	H	R
9	42	10.7	Cl	L
10	51	184.3	Cl	R
11	77	0.5	H	L
12	43	1.4	Cl	B
13	30	106.8	Cl	B
14	17	270.8	CY	B
15	77	109	CY	B
16	10.2	43	H	B
17	40	12.0	CY-liver	R
18	18	12.4	Cl	B
19	56	13.7	Cl	R
20	31	13.8	H	B
21	55	13.9	Cl	L
22	68	14.1	CY	B
23	97	14.5	Cl	B
24	29	15.0	H	L
25	43	15.4	H	B
26	52	15.4	CY-lymph node	B
27	27	15.8	Cl	B
28	52	16.2	Cl	R
29	44	16.8	Cl	R
30	45	17.8	CY-liver	B
31	106	18.0	H	B
32	43	19.4	H	B
33	49	2.7	CY	L
34	24	2.8	H	L
35	54	25.6	H	R
36	16	25.7	Cl	R
37	41	26.3	Cl	R
38	32	61.7	H	R
39	26	79.7	H	B
40	46	8.3	Cl	R
41	52	89.8	Cl	L
42	16	25.3	H	R
43	30	106.8	Cl	L
44	25	13.3	Cl	L
45	39	3.2	CY	B
46	26	30.2	Cl	R
47	65	30.3	H	R
48	65	31.1	CY-lymph node	B
49	28	36.0	CY-liver	B
50	48	38.8	CY-lymph node	B
51	40	4.0	Cl	R
52	61	4.8	H	R
53	40	41.8	Cl	B
54	41	42.1	CY	R
55	31	42.8	CY-liver	R
56	35	43.4	CY	R
57	38	45.4	Cl	B
58	62	5.0	Cl	B
59	42	5.3	CY	B
60	54	5.6	CY	B
61	55	5.6	H	R
62	3	5.8	Cl	B
63	46	51.6	CY	R
64	39	54.2	Cl	B
65	70	56.6	CY	L
66	35	6.1	Cl	L
67	43	6.2	H	L
68	37	6.3	Cl	B
69	45	6.6	Cl	R
70	29	72.3	CY	R
	*Normoglycemic subjects received corticosteroid treatment.			
